# Supplementary material for: Electrically responsive photonic crystals with bistable states for low-power electrophoretic color displays
Source: Nat Commun. 2022 Nov 16;13:7007. doi: 10.1038/s41467-022-34745-0 (PMC9669026; doi:10.1038/s41467-022-34745-0)
Supplement: Supplementary file 1 — Supplementary Information [file 41467_2022_34745_MOESM1_ESM.pdf]

Supplementary Information

Electrically Responsive Photonic Crystals with Bistable States for Low-power Electrophoretic Color Displays

*Qianqian Fu, Wenyuan Yu, Guangyang Bao, and Jianping Ge**

Supplementary Figures

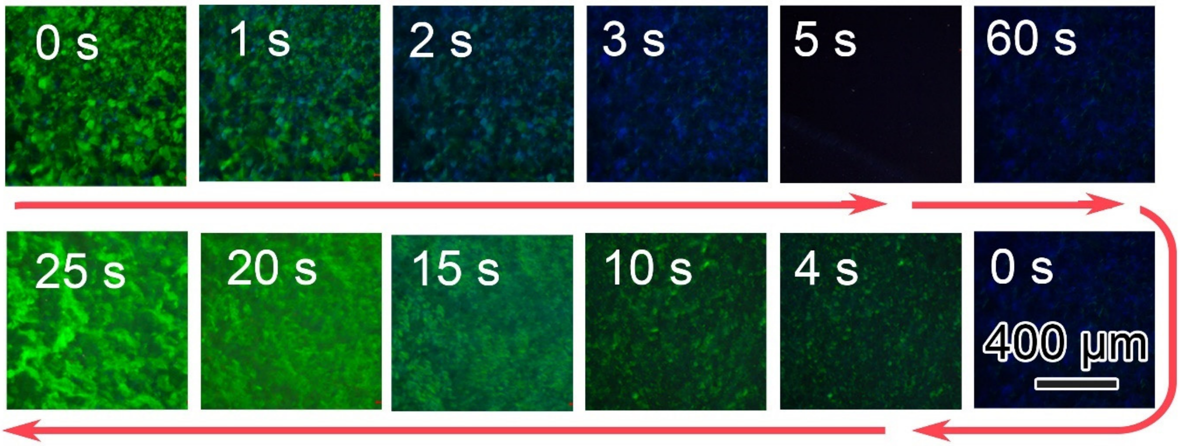

Supplementary Figure 1. OM images of SiO_2/PCb-PEG-EG ERPC during optical switching. Optical microscope (OM) images of the SiO_2/PCb-PEG-EG ERPC in the switching between the colored state and the colorless state.

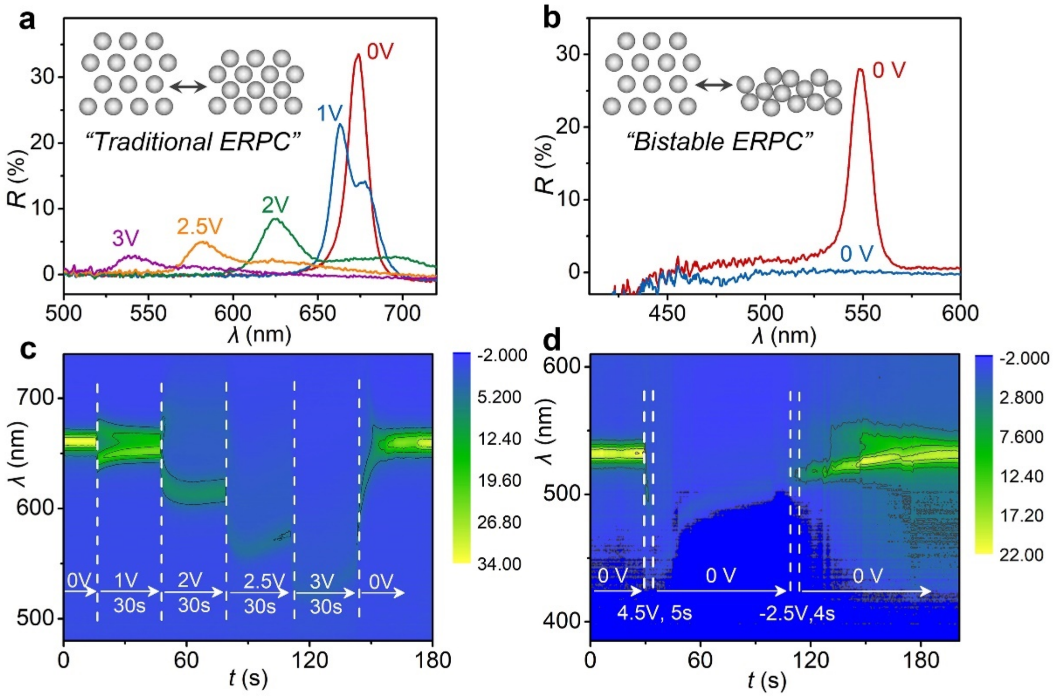

Supplementary Figure 2. Comparison of the traditional and the bistable ERPC. a Reflection spectra and **c** dynamic reflection spectra (DRS) for the traditional ERPC based on "lattice shrinkage and expansion". **b** Reflection spectra and **d** DRS pattern for the bistable ERPC based on "colloidal assembling and disassembling" under different E-field conditions. Here, "traditional ERPC" refers to the previously reported electrophoretic ERPC based on lattice shrinkage and expansion.

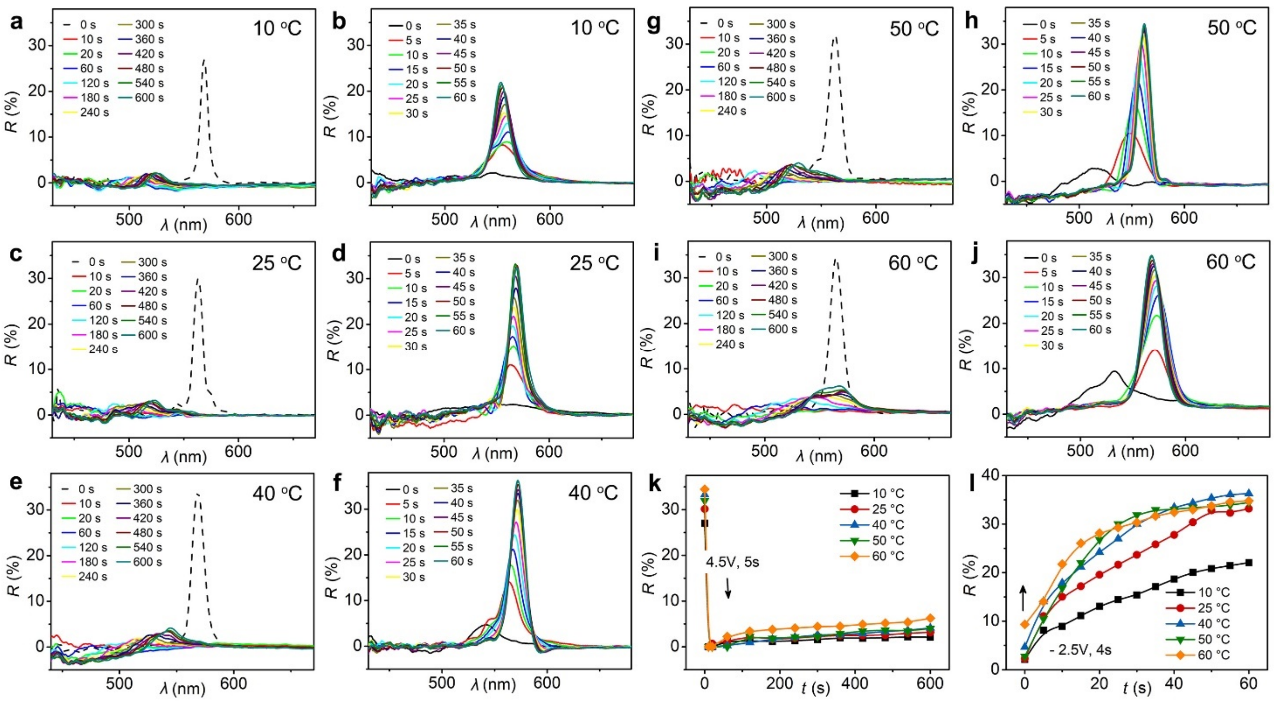

Supplementary Figure 3. Temperature influence on the bistable characteristics. The change of reflection spectra of SiO_2/PCb-PEG-EG ERPCs at **a**, **b** 10 °C, **c**, **d** 25 °C, **e**, **f** 40 °C, **g**, **h** 50 °C, **i, j** 60 °C. Corresponding time evolution of reflection intensities when **k** 4.5V was applied to the ERPC for 5s to turn it from the colored state to the colorless state, and **l** -2.5V was applied to the ERPC for 4s to turn it from the colorless state to the colored state.

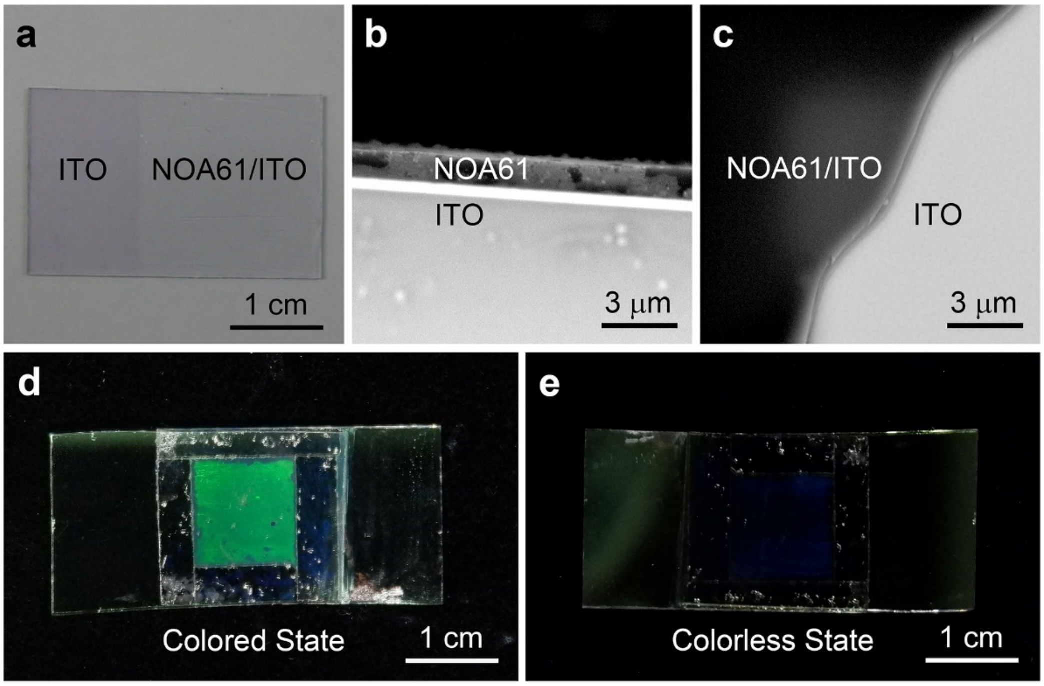

Supplementary Figure 4. Electrophoretic mechanism proved by the electrode passivation. a Digital photos, **b** cross-sectional SEM image, and **c** top-view SEM image of NOA61-coated ITO glass. **d** The colored and **e** the colorless state of the bistable ERPC sealed between a NOA61-coated ITO and an untreated ITO electrode. The passivation of electrode by NOA 61 and yet the retaining of bistability property exclude the "electrochemical mechanism" and support the "electrophoretic mechanism".

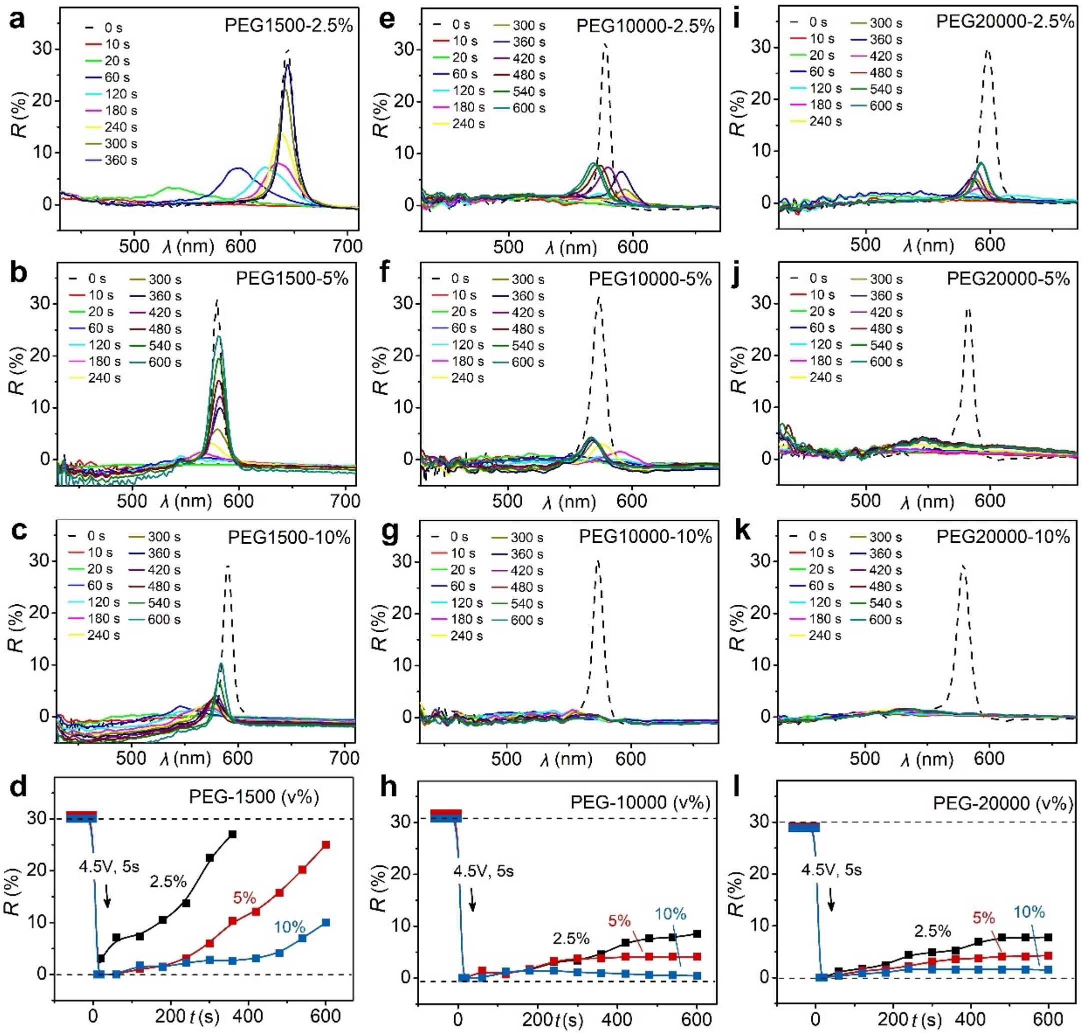

Supplementary Figure 5. Influence of PEG molecular weight and volume fraction on the holding of colorless state. The change of reflection spectra of SiO_2/PCb-PEG ERPCs with different volume fraction of **a-c** PEG1500, **e-g** PEG10000, and **i-k** PEG20000, and **d**, **h**, **l** the corresponding time evolution of reflection intensities when 4.5V was applied to the ERPC for 5s to turn it from the colored state to the colorless state. The dash curves indicated the reflection signal of the colored state.

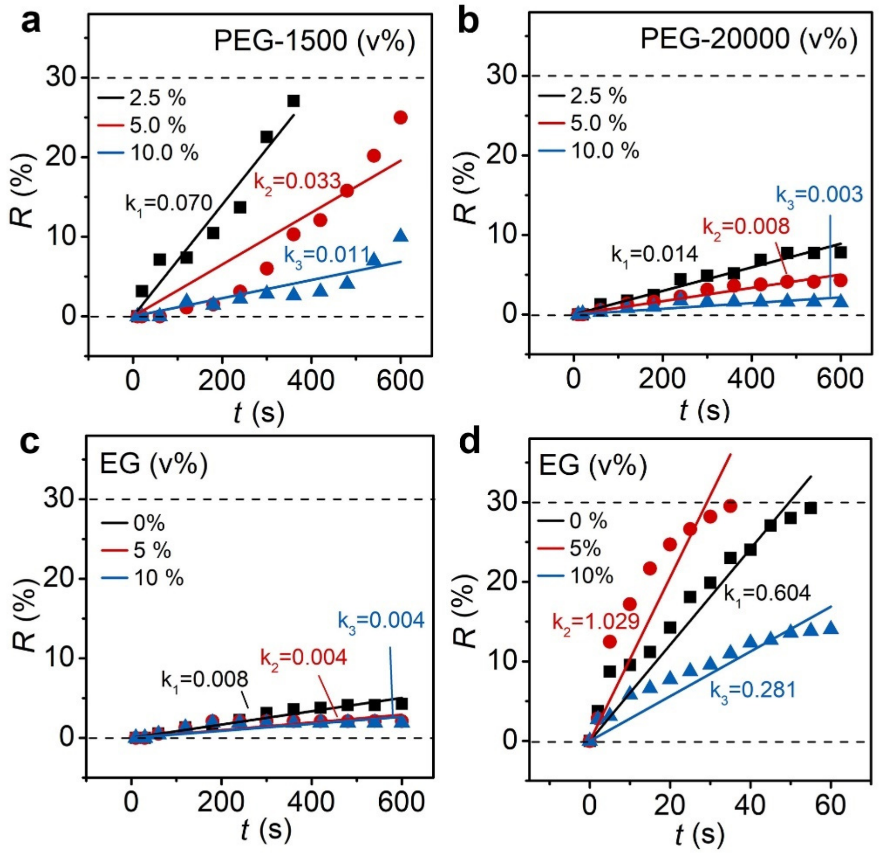

Supplementary Figure 6. Quantification of the recovery rate of reflection intensity ($\Delta R/t$). a-c Linear fitting of the time evolution of reflection intensity after the exertion of 4.5V for 5s or **d** -2.5V for 4s. The "*R-t*" curves are fitted in a linear way, because the slopes ($\Delta R/t$) can easily reveal the recovery rate of the colored states. Although there are deviations in **a** and **d**, it won't affect the judgement about the influence of PEG or EG volume fraction upon the recovery rate.

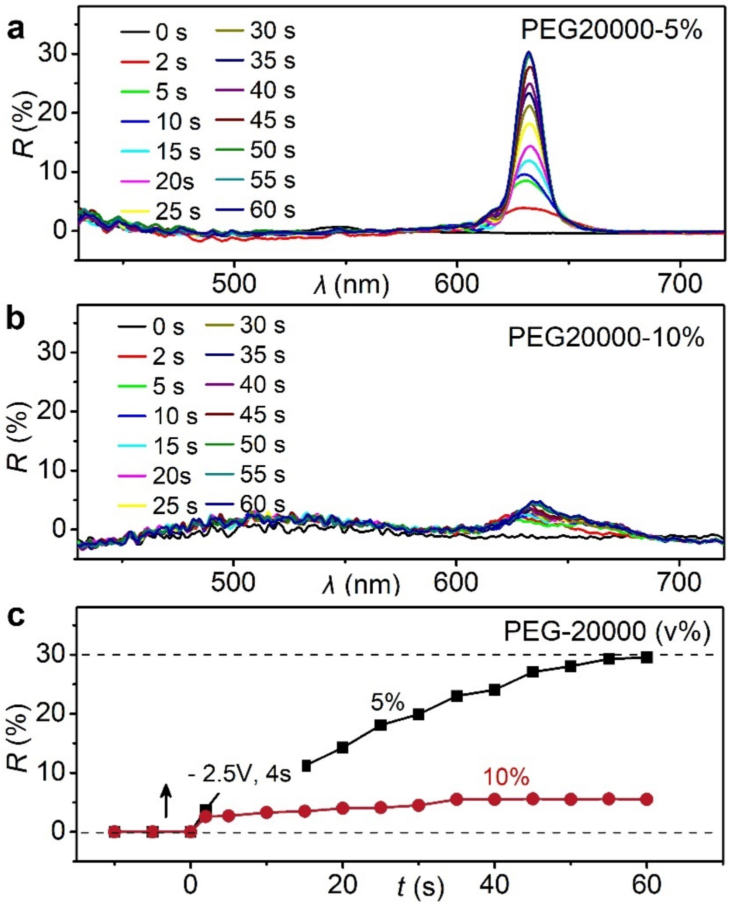

Supplementary Figure 7. Influence of PEG volume fraction on the recovery of colored state. The change of reflection spectra of SiO_2/PCb-PEG20000 ERPCs with PEG fraction of **a** 5% and **b** 10%, and **c** the corresponding time evolution of reflection intensity after -2.5V was applied to the ERPC for 4s to turn it from the colorless state to the colored state.

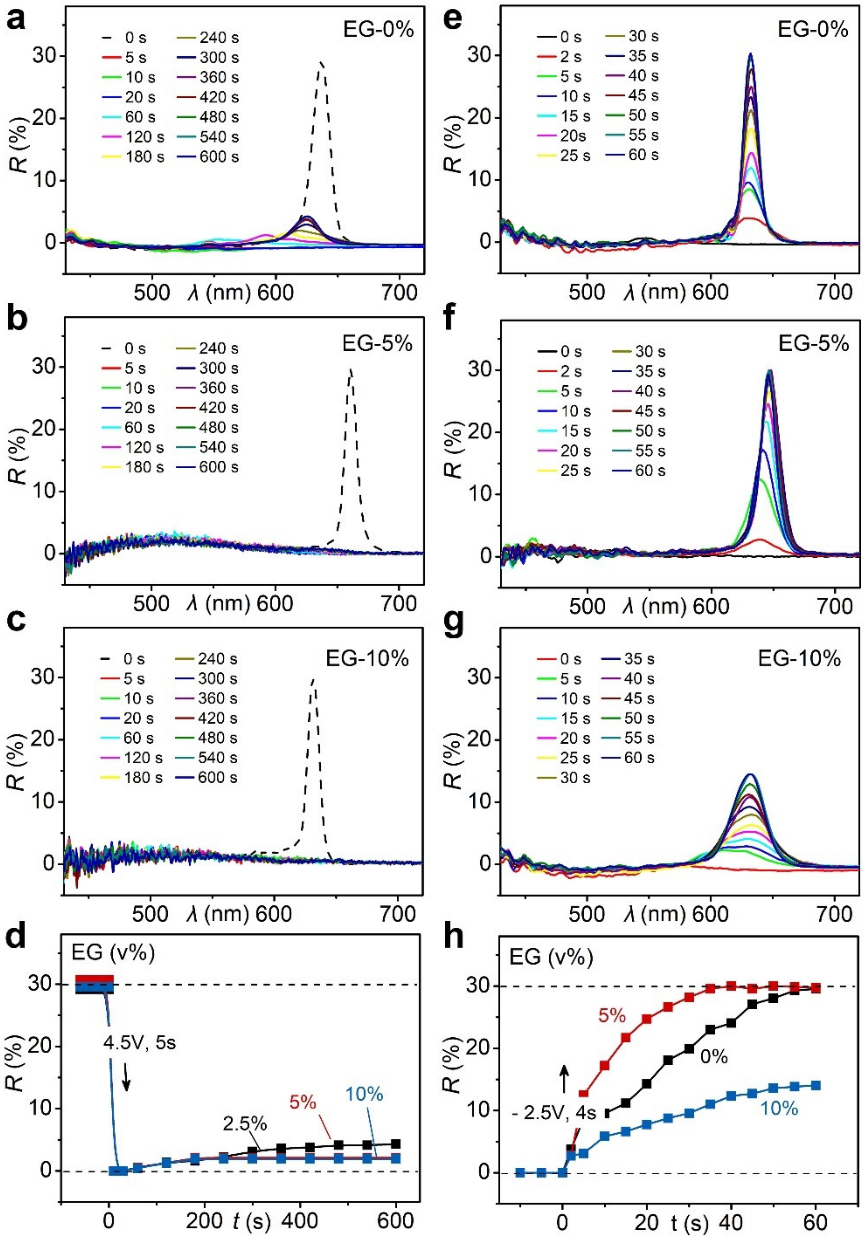

Supplementary Figure 8. Influence of EG volume fraction on both the holding of colorless state and the recovery of colored state. a-c The change of reflection spectra of SiO_2/PCb-PEG20000-EG ERPCs with different fraction of EG, and **d** the corresponding time evolution of reflection intensities when holding the colorless state. The dash curves indicated the reflection signal of the colored state. **e-g** The change of reflection spectra with time and **h** the corresponding evolution of reflection intensities during the recovery of the colored state.

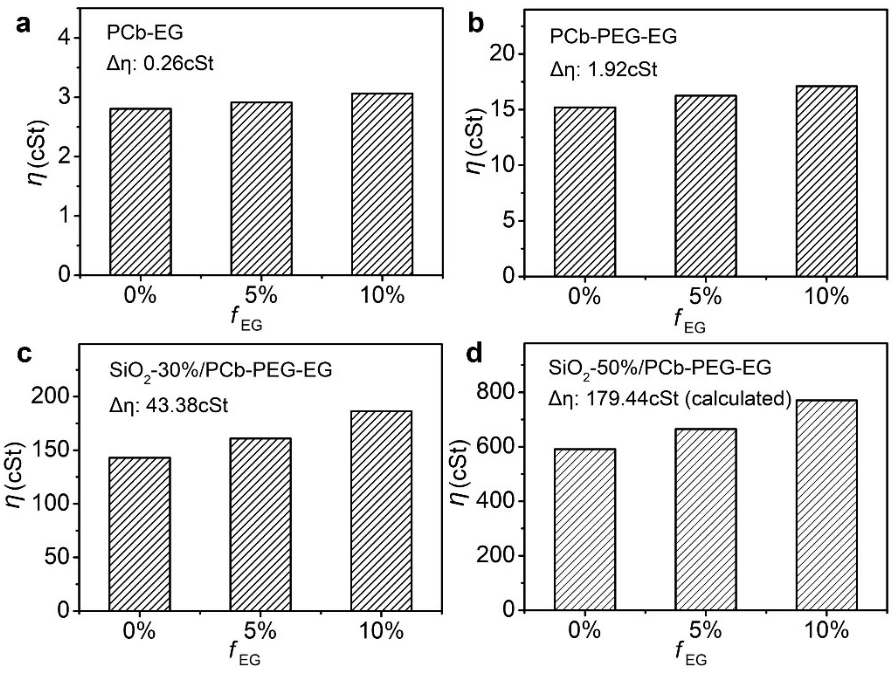

Supplementary Figure 9. Viscosity evolution with f_{EG}. a-c viscosities of PCb-EG, PCb-PEG-EG, and SiO_2-30%/PCb-PEG-EG solutions measured by the viscometer, and **d** viscosity of SiO_2-50%/PCb-PEG-EG solutions calculated from that of SiO_2-30%/PCb-PEG-EG solutions

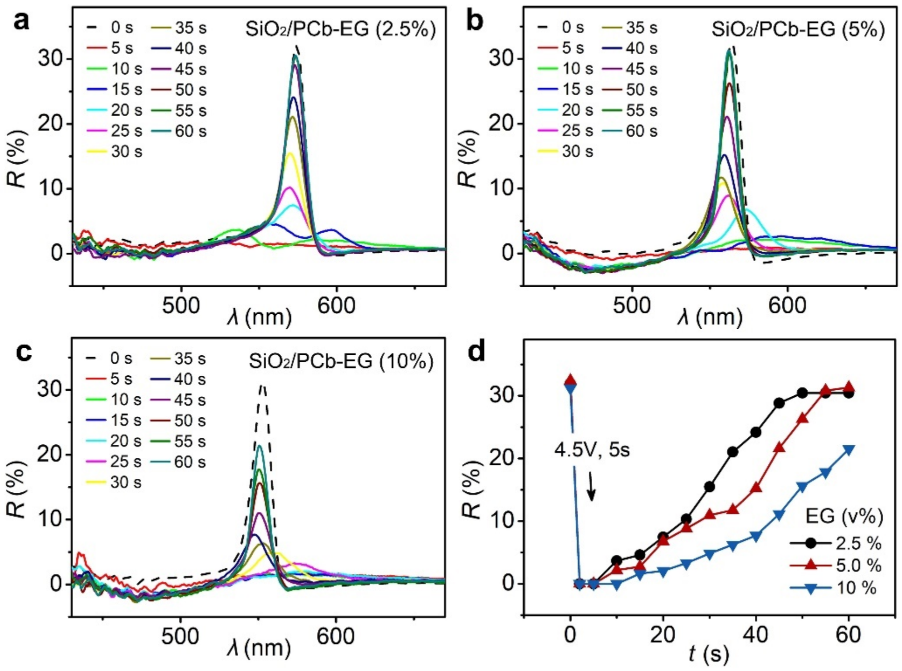

Supplementary Figure 10. Unstable disordered state for ERPC without PEG. The change of reflection spectra of SiO_2/PCb-EG ERPCs with f_{EG} of **a** 2.5%, **b** 5%, **c** 10%, and **d** the corresponding time evolution of reflection intensities after 4.5V was applied to the ERPC for 5s.

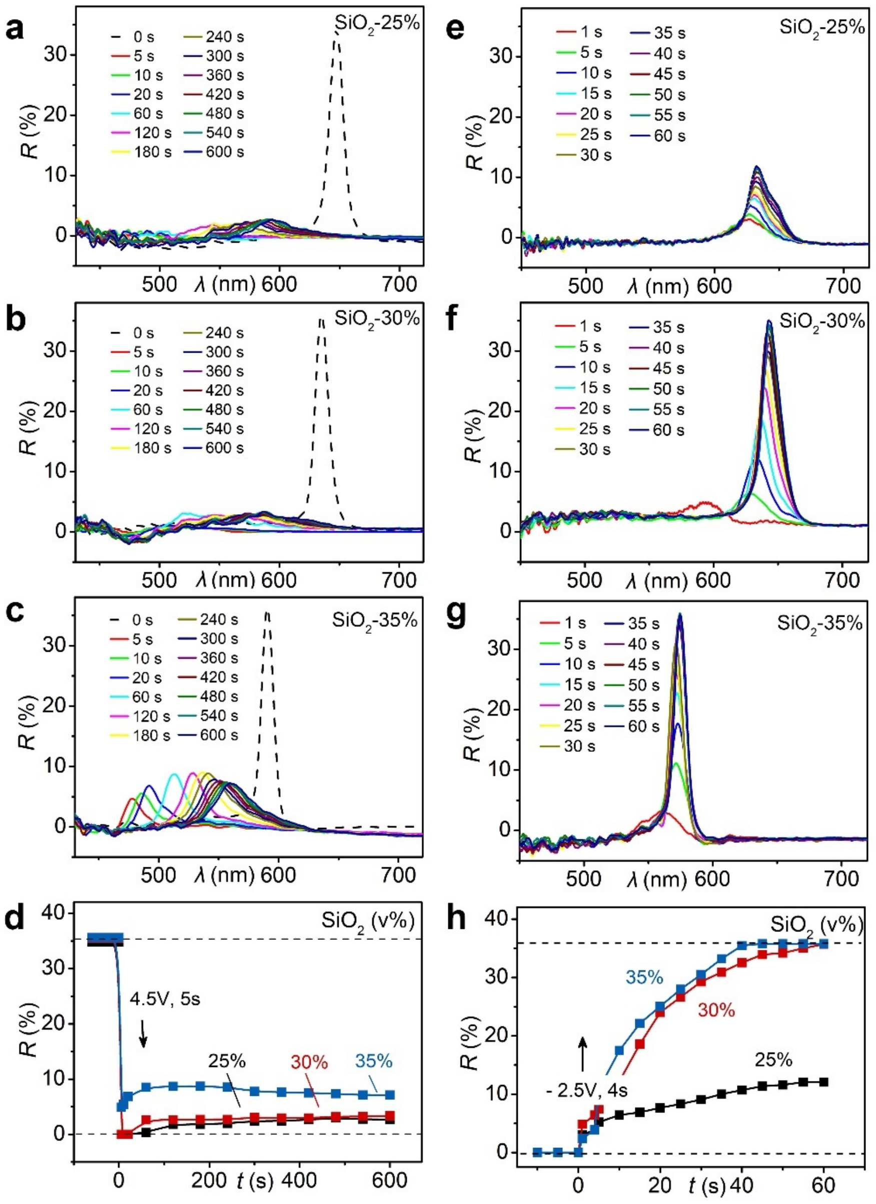

Supplementary Figure 11. Influence of SiO_2 volume fraction on both the holding of colorless state and the recovery of colored state. a-c The change of reflection spectra of SiO_2/PCb-PEG20000-EG ERPCs with different fraction of SiO_2 particles, and **d** the corresponding time evolution of reflection intensities when holding the colorless state. The dash curves indicated the reflection signal of the colored state. **e-g** The change of reflection spectra with time and **h** the corresponding evolution of reflection intensities during the recovery of the colored state.

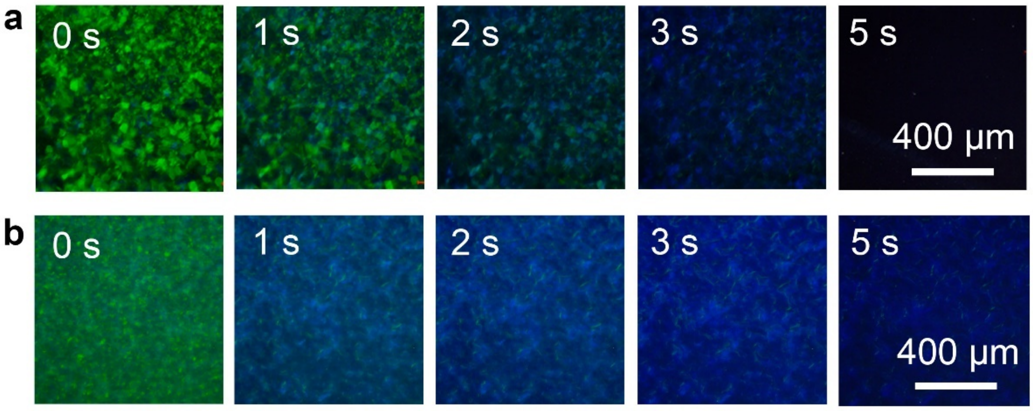

Supplementary Figure 12. Influence of SiO_2 volume fraction on the holding of colorless state studied by OM images. Optical microscope images of the SiO_2/PCb-PEG20000-EG ERPCs with SiO_2 fraction of **a** 30% and **b** 35% after 4.5V was applied to the ERPC for 5s to turn it from the colored state to the colorless state. The images shown in **a** and **b** are always showing the same sample area. Figure **b** suggested that the ERPC with f_{SiO2} of 35% was difficult to be switched to the colorless state due to its strong tendency to form colloidal crystals.

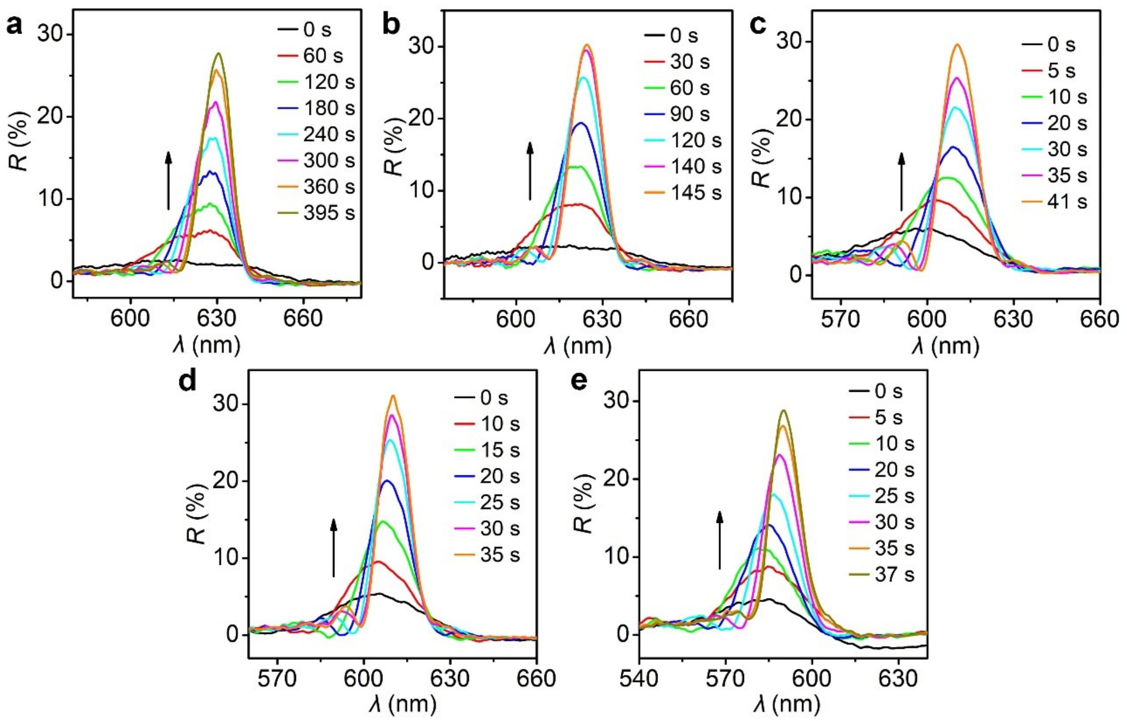

Supplementary Figure 13. High SiO_2 volume fraction accelerate the colloidal assembly in the absence of electric field. The change of reflection spectra during the self-assembly of the SiO_2/PCb-PEG20000-EG colloidal crystals with f_{SiO2} of **a** 25%, **b** 27.5%, **c** 30%, **d** 32.5%, and **e** 35%. These spectra indicated that colloidal solution with high f_{SiO2} would accelerate the colloidal assembly in the absence of electric field.

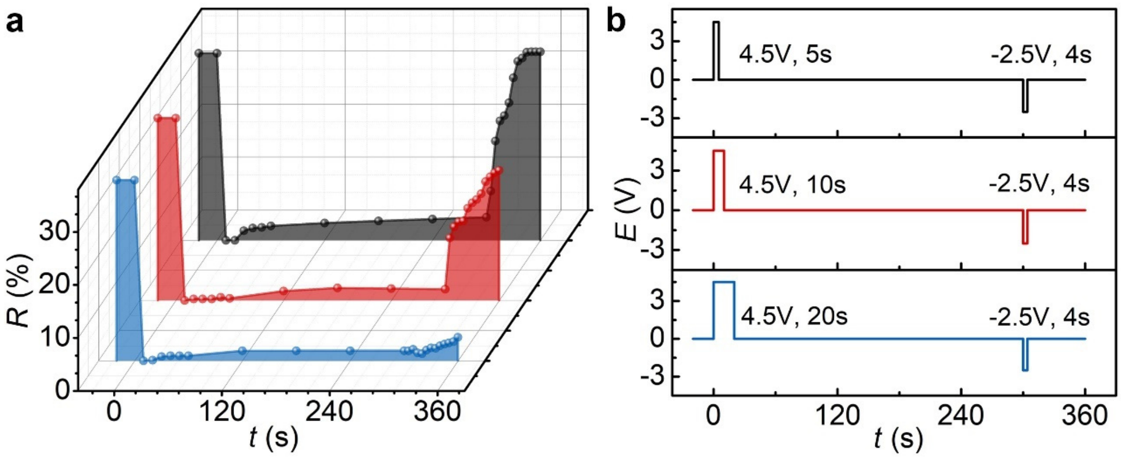

Supplementary Figure 14. Influence of forward voltage duration on the holding of colorless state and the recovery of colored state. a Time evolution of reflection intensities of the SiO_2/PCb-PEG-EG ERPC under **b** a series of electric field waveforms with the forward voltage duration time being set as 5 s, 10 s, and 20 s. The time evolutions of reflection intensity plotted in black, red, and blue curve in **a** were recorded under the application of E-fields in **b** indicated by the same color.

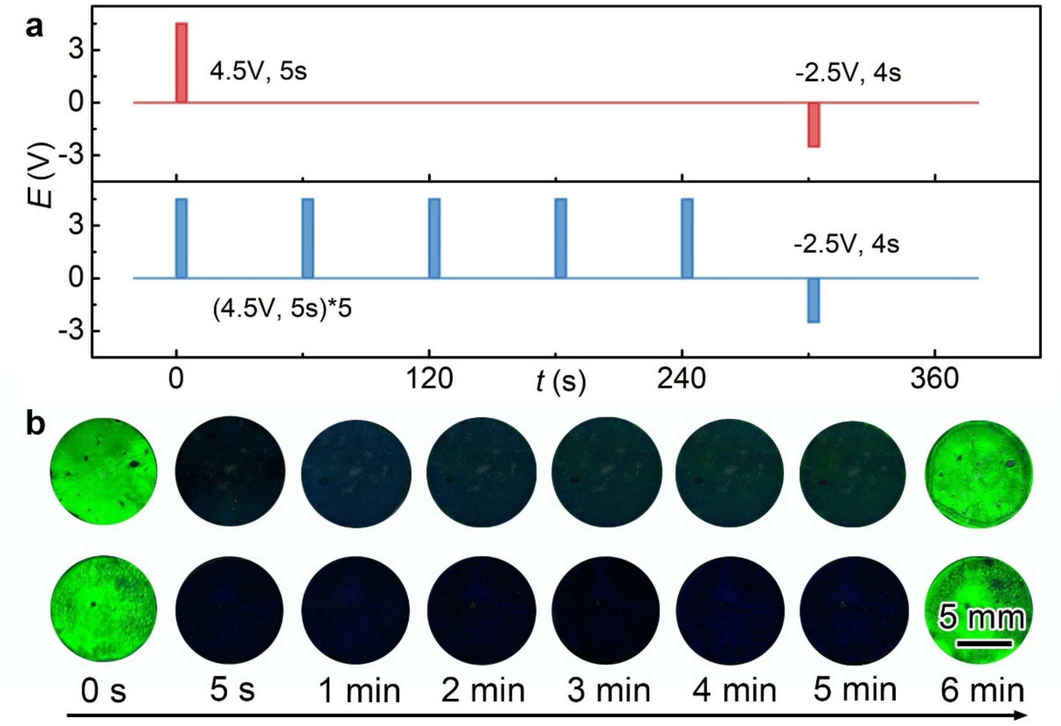

Supplementary Figure 15. Better holding of the colorless state through intermittent E-field exertion. a The application of a single forward voltage of 4.5V (red plots) and intermittent voltages with interval of 60s (blue plots) to the ERPC, and **b** the resulting switching between the colored and the colorless state.

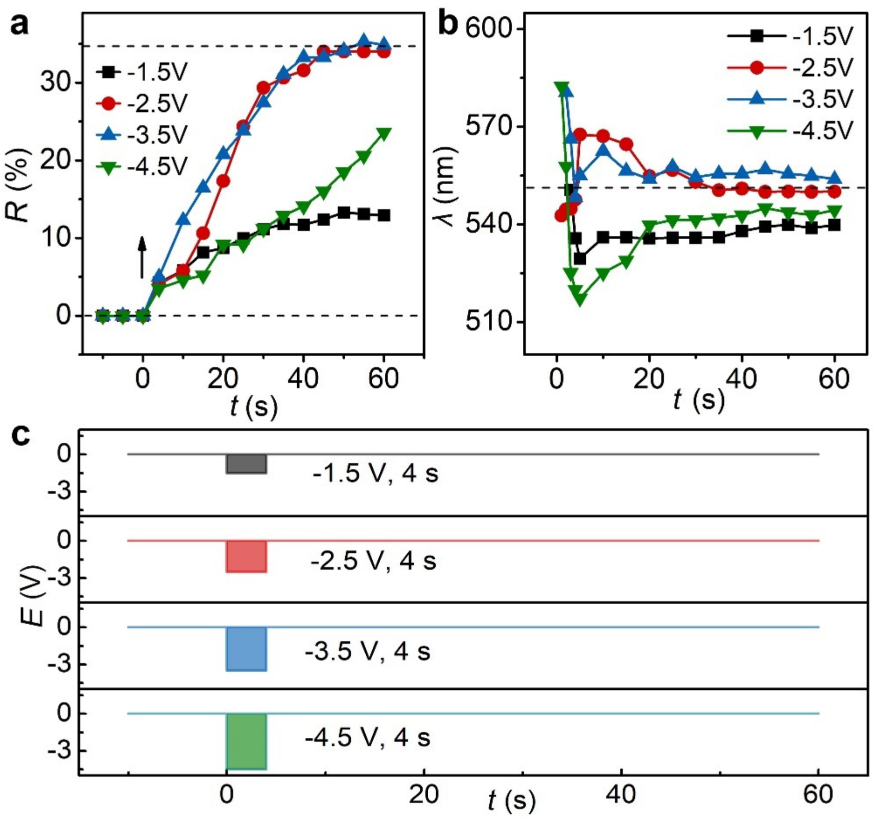

Supplementary Figure 16. Influence of inverse voltage strength on the recovery of colored state. **a**, **b** The change of reflection intensities and wavelengths for the SiO_2/PCb-PEG-EG ERPC after **c** a group of inverse voltages with different strength (-1.5 V to -4.5 V) were applied to recover the ERPC to the colored state. The time evolution of R and λ plotted in black, red, blue, and green curves in **a**, **b** were recorded under the corresponding E-fields in **c** indicated by the same color. The dash lines in **a** indicated the reflection intensity of ERPC in two states. The dash line in **b** indicated the expected reflection wavelength for the colored state. It should be noted that the λ after application of -4.5V was below the expected value, because a strong inverse voltage first pull the particles away from each other to expand (recover) the PC lattice and then compress the lattice towards the opposite electrode.

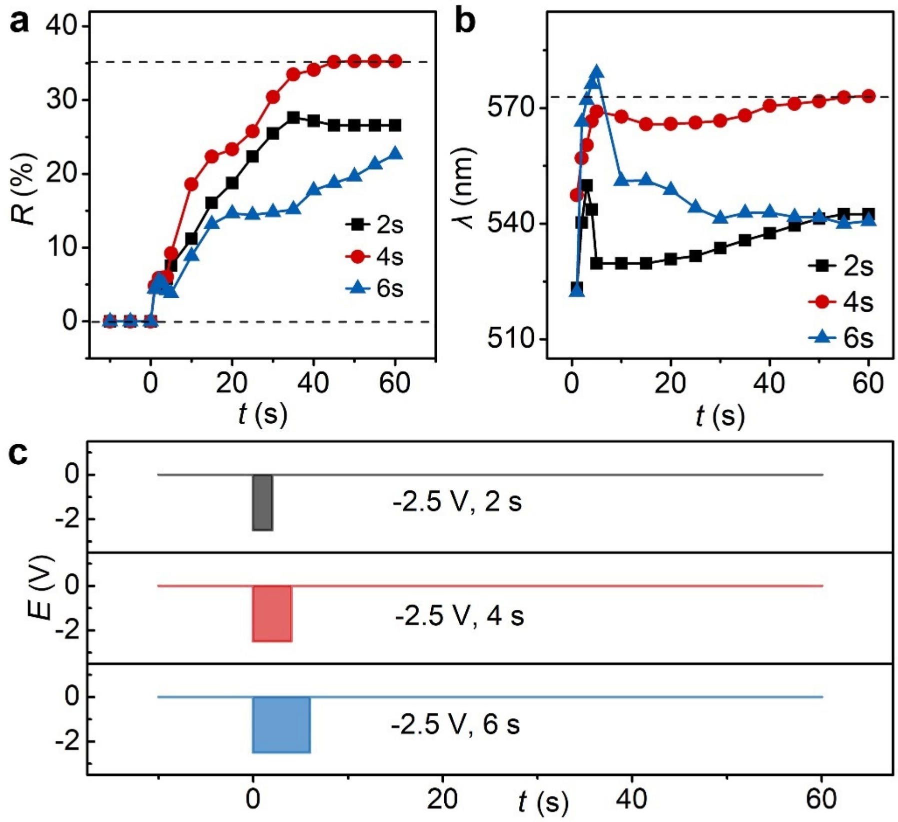

Supplementary Figure 17. Influence of inverse voltage duration on the recovery of colored state. **a**, **b** The change of reflection intensities and wavelengths for the SiO_2/PCb-PEG-EG ERPC after **c** a group of inverse voltages with different duration (2 s to 6 s) were applied to recover the ERPC to the colored state. The time evolution of R and λ plotted in black, red, and blue curves in **a**, **b** were recorded under the corresponding E-fields in **c** indicated by the same color. The dash lines in **a** indicated the reflection intensity of ERPC in two states. The dash line in **b** indicated the expected reflection wavelength for the colored state. It should be noted that the λ after application of -2.5V for 4s/6s was below the expected value, because a long exertion of inverse voltage first pull the particles away from each other to expand (recover) the PC lattice and then compress the lattice towards the opposite electrode.

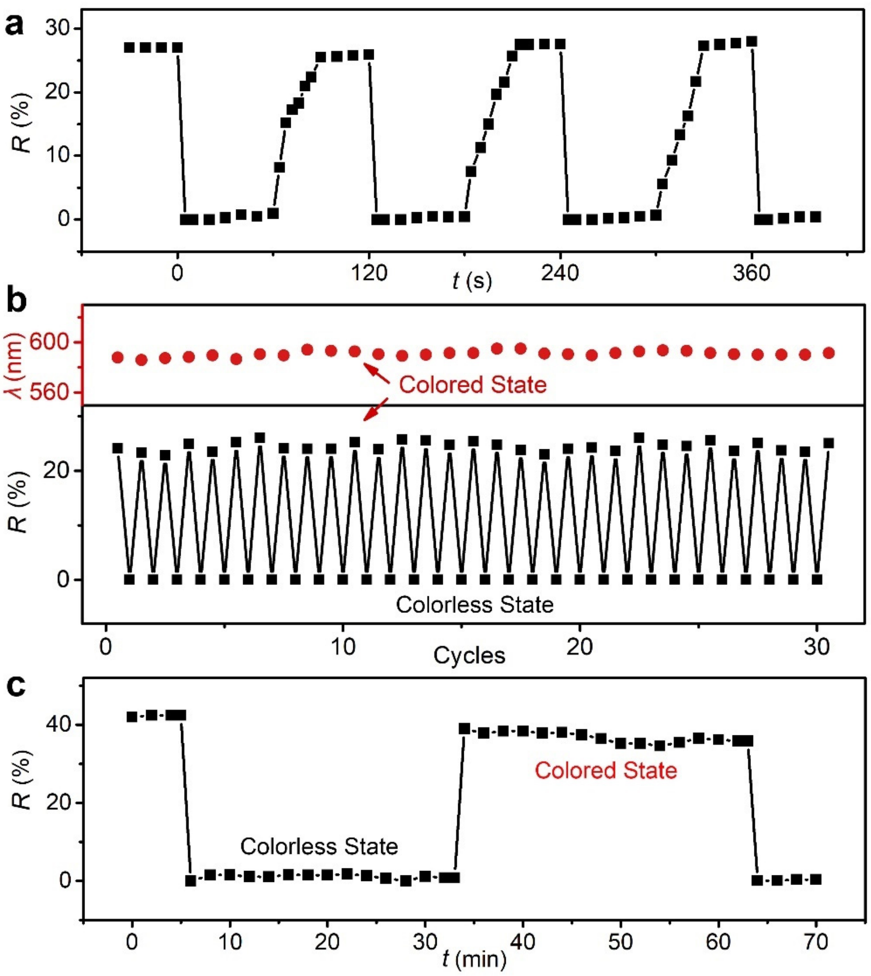

Supplementary Figure 18. Reversible switching of the bistable ERPC. a Time evolution of reflection intensity during the switching of the colored and colorless state in a period of 60 s, and **b** the change of λ and R in an extended test with 30 cycles of switching. **c** Time evolution of reflection intensity in the switching in a period of 30 min.

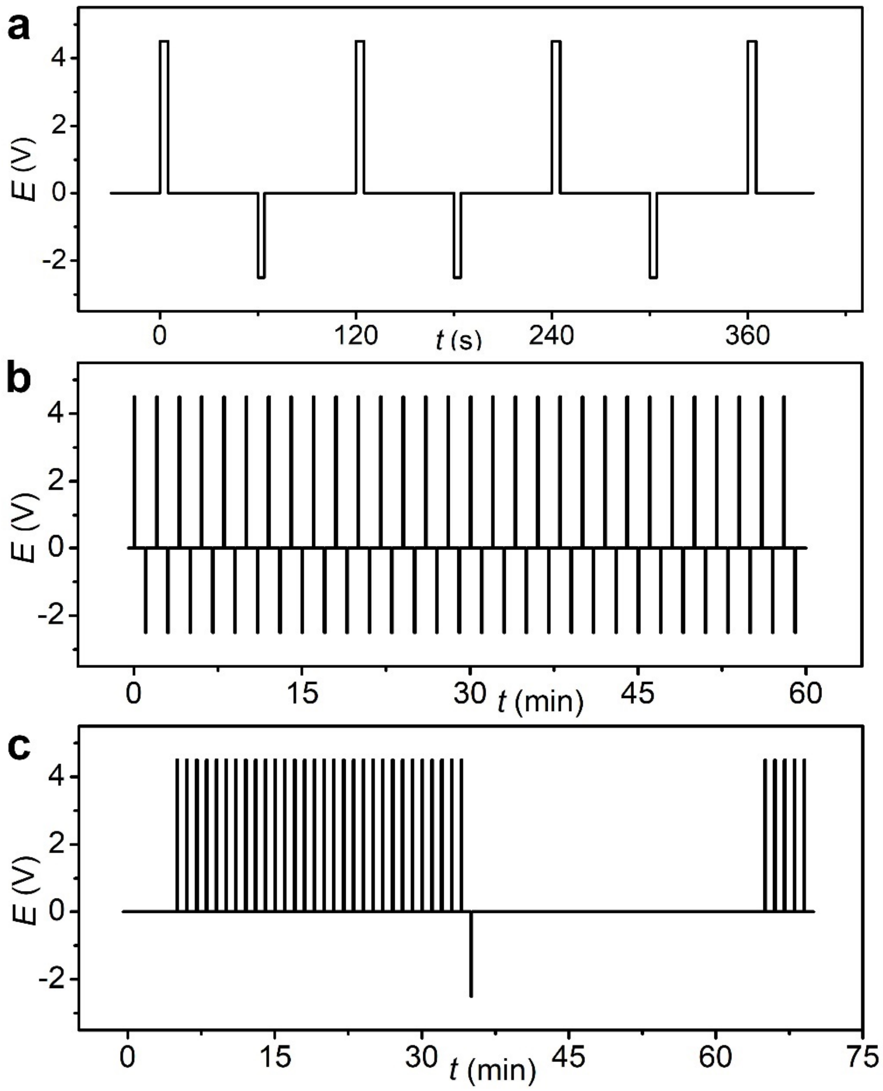

Supplementary Figure 19. Electric filed waveforms for the reversible switching of the bistable ERPC. The electric field waveforms used to switch the bistable ERPC in a period of **a**, **b** 60 s and **c** 30 min. The corresponding change of the reflection signals were presented above.

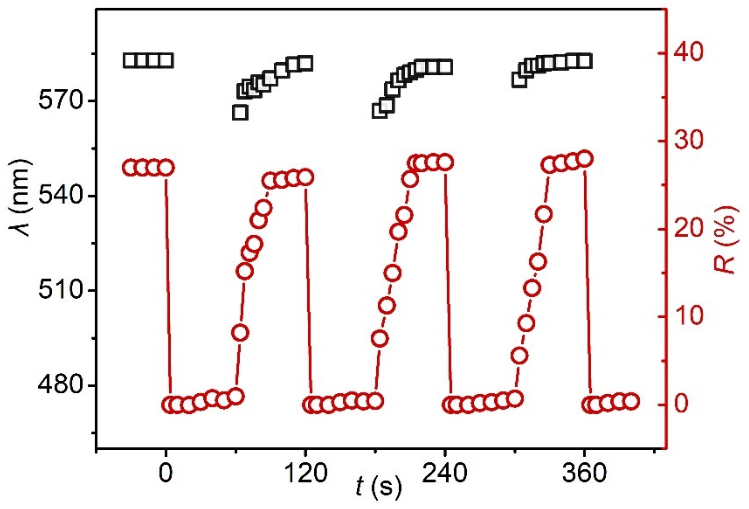

Supplementary Figure 20. Supplied change of reflection wavelength during the switching of bistable ERPC. The supplied change of the λ corresponding to the change of intensity during the switching between two states. The switching from the colored to the colorless state takes several seconds. It should be noted that the perceptible switching from the colorless to the colored state also takes several seconds, because the λ showed little changes during the recovery of full reflection intensity.

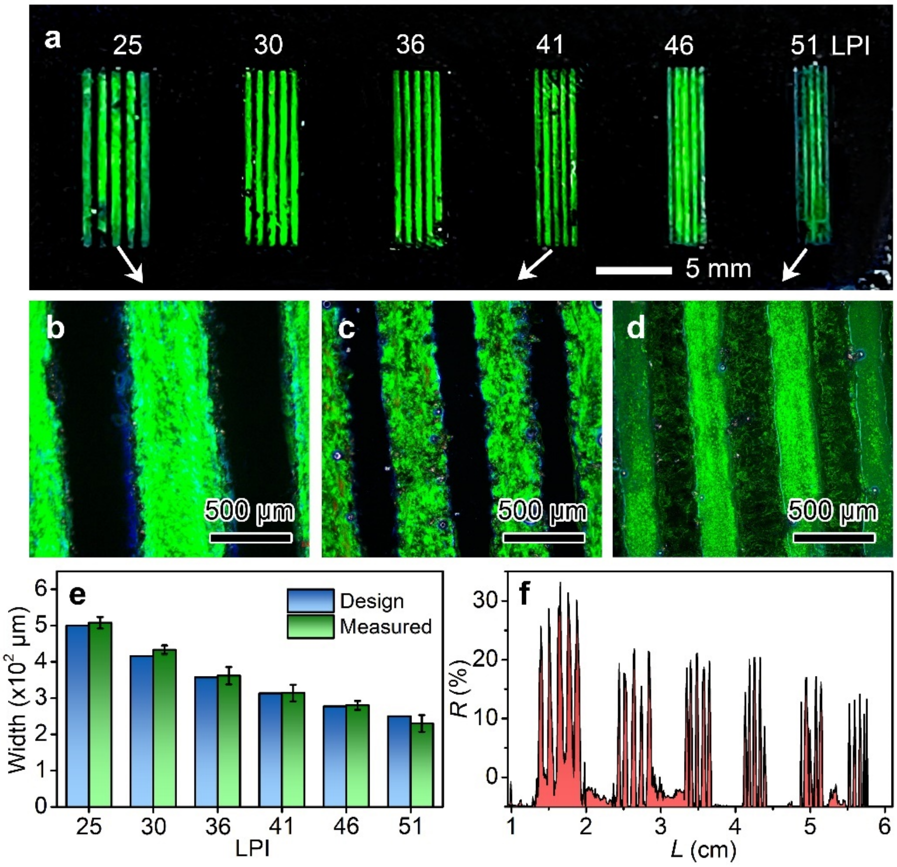

Supplementary Figure 21. High-resolution display based on micron-scale cells. a Digital photos and **b-d** OM images of the green ERPC encapsulated in the strip-like cells with different width; **e** comparison of the designed and measured width of the display unit, and the practical resolution (LPI) considering the width of cells and intervals; **f** evolution of reflection intensity as the microscopic spectrometer horizontally scanning across the display units.

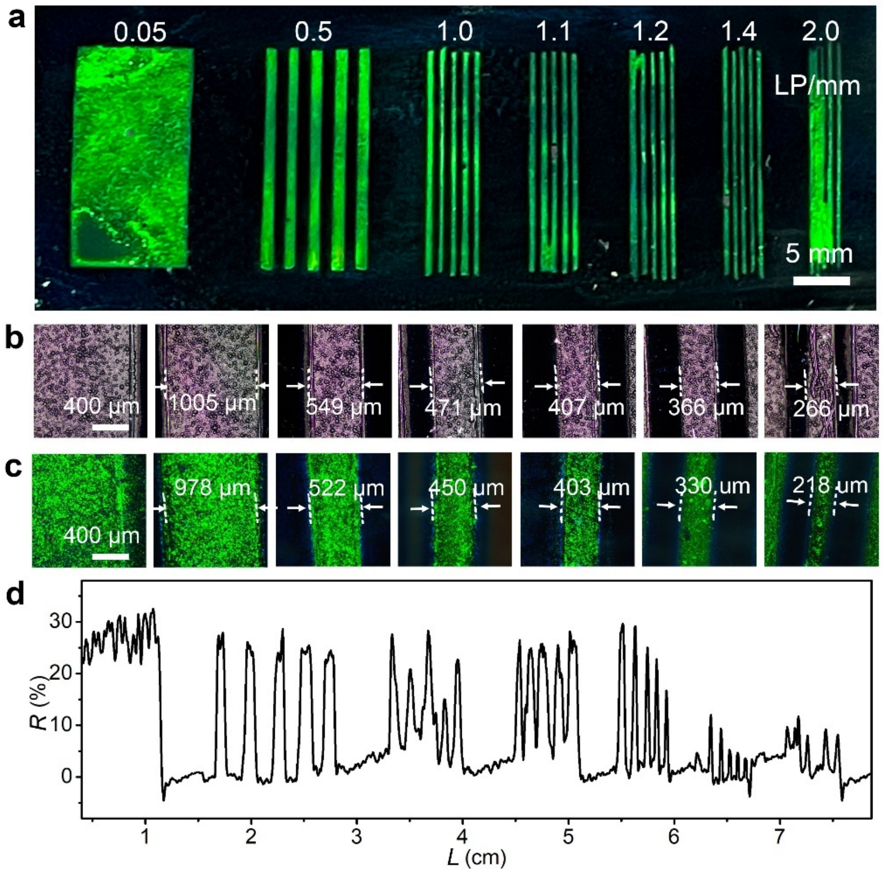

Supplementary Figure 22. High-resolution display realized by patterned electrodes. a Digital photos of a group of green ERPC lines with different widths, which was displayed by the exertion of 4.5 V to the patterned electrode; **b** OM images of the insulated lines on ITO glass and **c** the resulting green PC lines after the exertion of 4.5 V; **d** evolution of reflection intensity as the microscopic spectrometer horizontally scanning across the ERPC patterns.

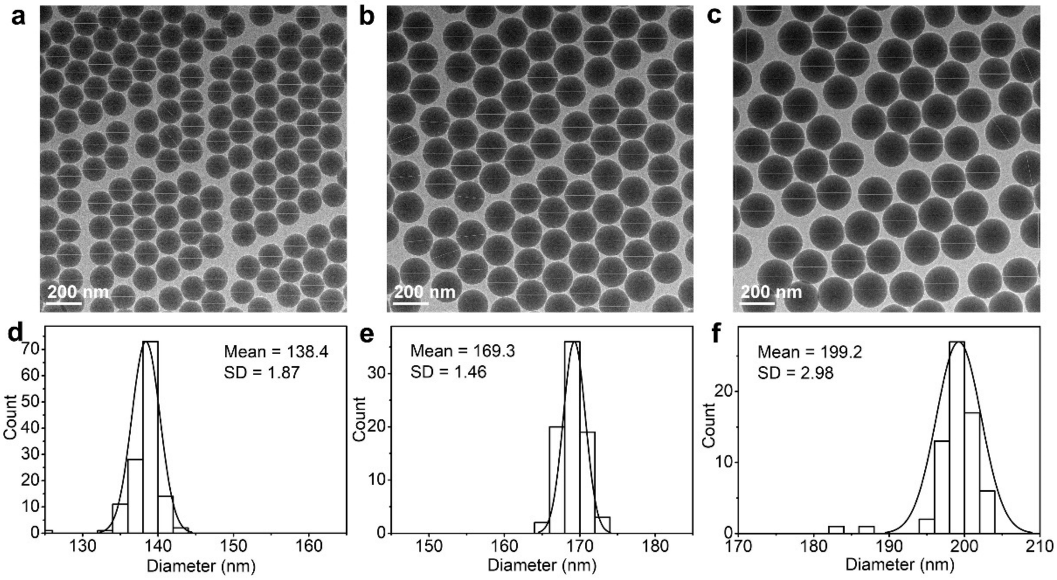

Supplementary Figure 23. SiO_2 particles for RGB ERPCs. TEM images and size distribution of the SiO_2 particles for preparing the **a**, **d** blue, **b**, **e** green, and **c**, **f** red ERPCs.

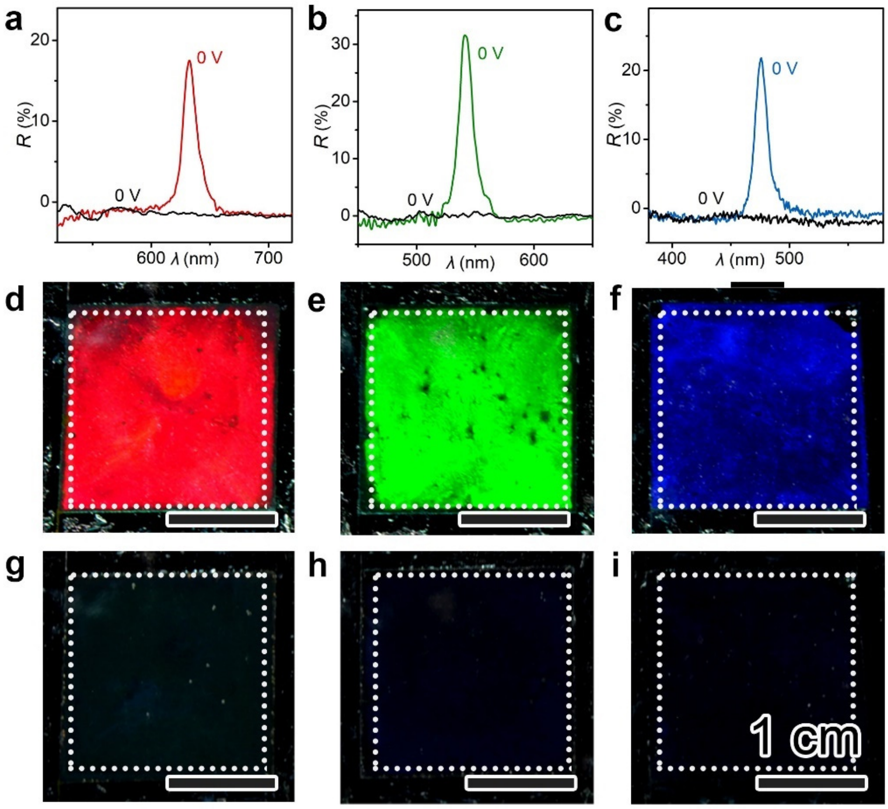

Supplementary Figure 24. Bistable ERPCs with RGB colors. a-c The “colored” and the black plots showed the reflection spectra of the red, green, and blue ERPCs in the colored and colorless state. **d-i** Corresponding digital photos of these ERPCs at two stable states.

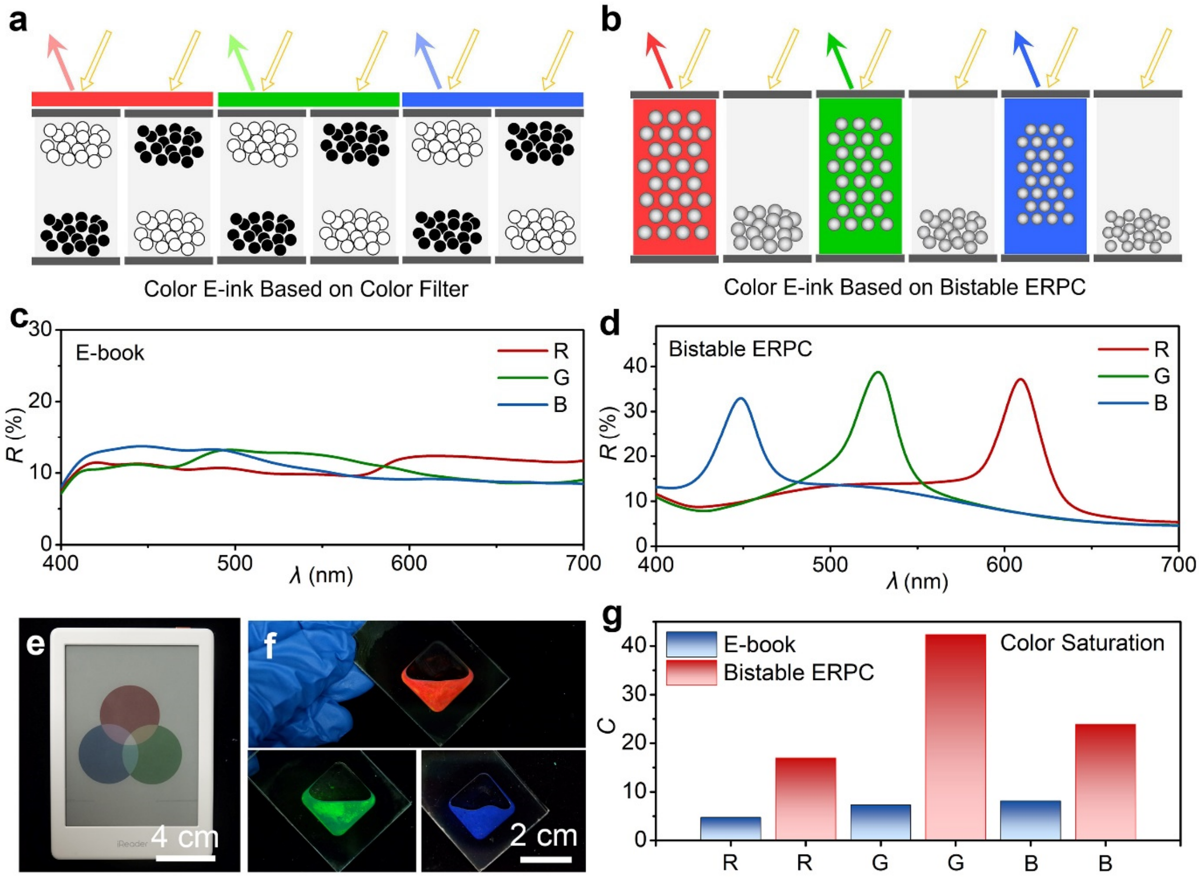

Supplementary Figure 25. The "color-filter" vs. the "bistable ERPC" electrophoretic display. a, **b** working mechanism, **c**, **d** reflection signals, **e**, **f** visual appearance, and **g** color contrast for the color electrophoretic display based on color-filter and bistable ERPC. Here, **c**, **d** the reflection spectra and **g** the color saturation were all measured by a colorimeter equipped with an integrating sphere. The sample was illuminated by a D65 light source with diffused illumination, and the reflected signals were collected by the integration sphere to output the color space parameters as well as the reflection spectra ranging from 400 nm to 700 nm with an interval of 10 nm.

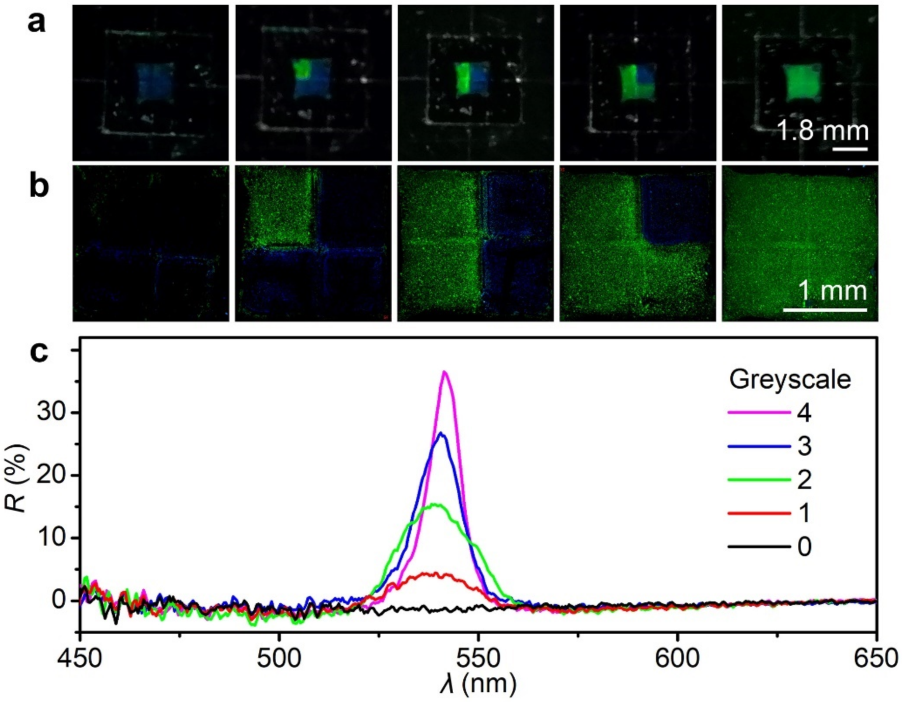

Supplementary Figure 26. Greyscale controlled by subpixel density. **a** Digital photos, **b** optical microscope images, and **c** reflection spectra of green ERPC with 4 different greyscales controlled by the density of colored subpixels.

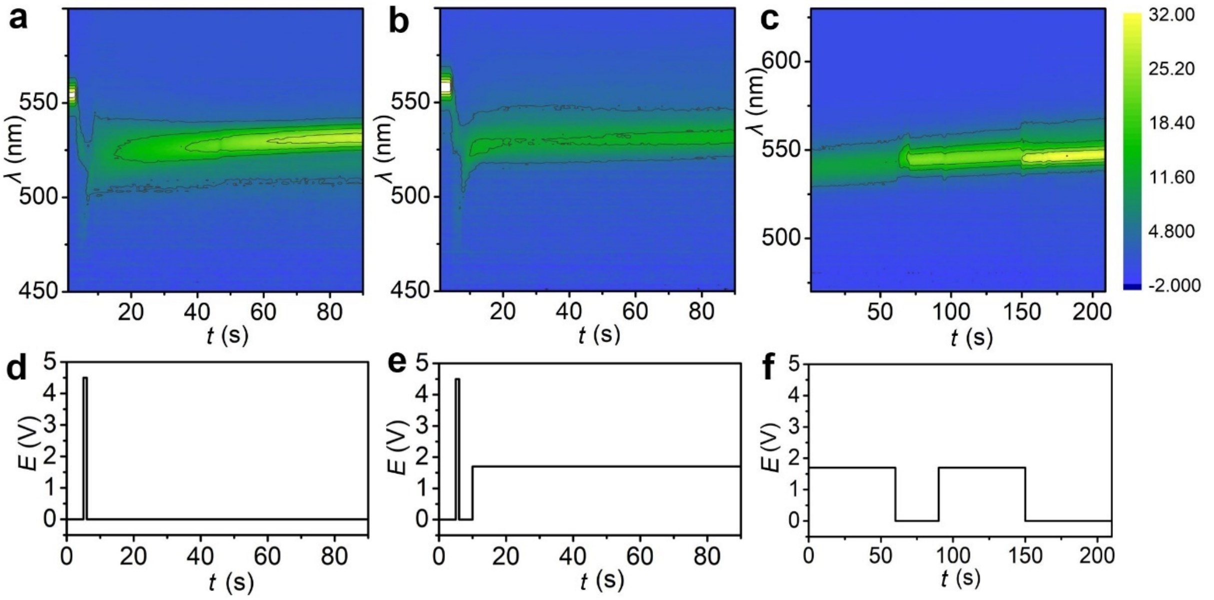

Supplementary Figure 27. Reflection intensity maintained by the bias voltage. a Dynamic reflection spectra (DRS) and **d** E-field modulation for a green ERPC showing the gradual recovery of reflection intensity after 4.5V was applied for 1s. **b** A specific reflection intensity can be maintained **e** when a bias voltage of 1.7 V is applied during the recovery process; **c** 3 kinds of reflection intensities realized by **f** the programmed application of bias voltage. Three DRS patterns shared the same color scales as listed in **c**.

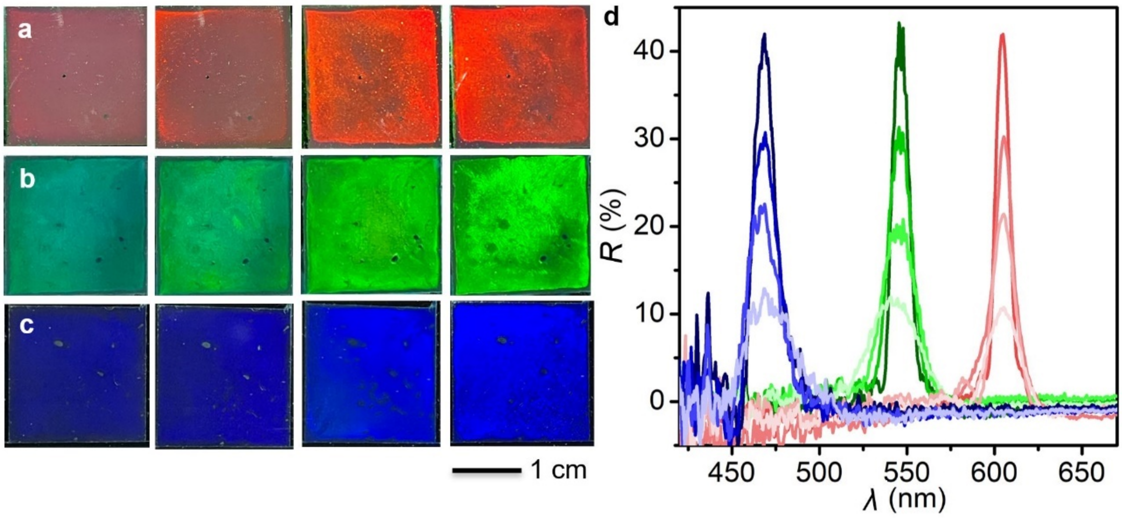

Supplementary Figure 28. Greyscale controlled by reflection intensity. a-c Digital photos and **d** reflection spectra of red, green, and blue ERPCs with 4 different greyscales controlled by the reflection intensity.

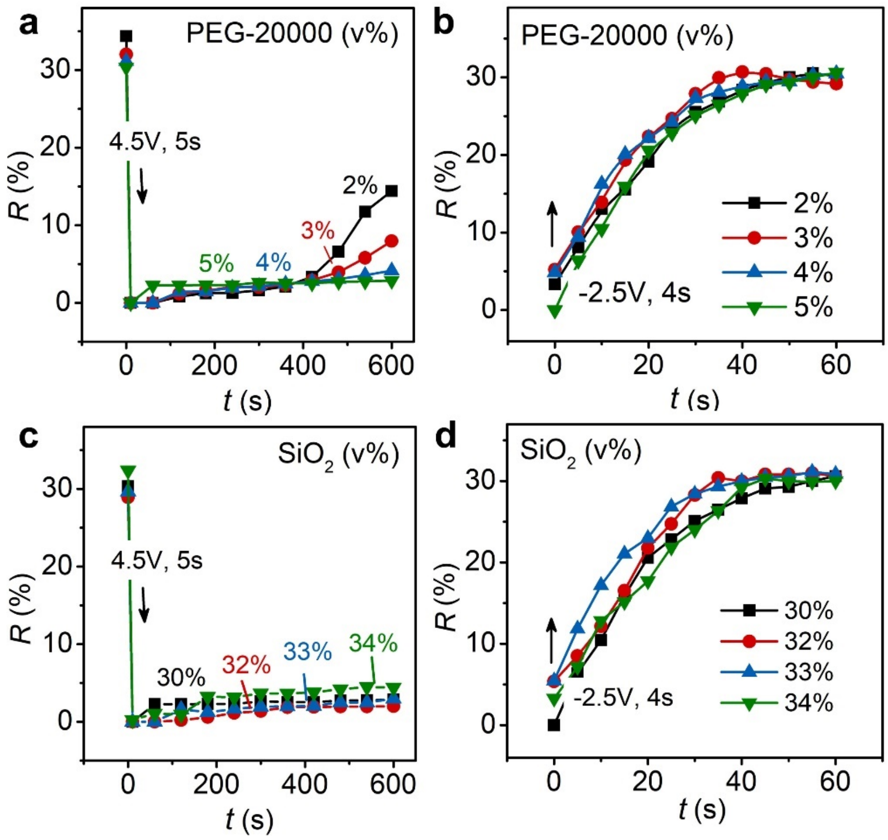

Supplementary Figure 29. Accelerating the recovery of colored state by tuning the ingredients of ERPC. a, **b** Influence of volume fraction of PEG-20000 on the holding of "colorless state" and recovery of "colored state", indicated by the time evolution of reflection intensities. When f_{PEG} decreased from 5% to 3%, the recovery time reduced from 38 s to 29 s under application of -2.5V for 4s. **c**, **d** Influence of volume fraction of SiO_2 particles on bistability. When the f_{SiO2} increased from 30% to 33%, the recovery time was shortened from 38 s to 27 s under application of -2.5V for 4s.

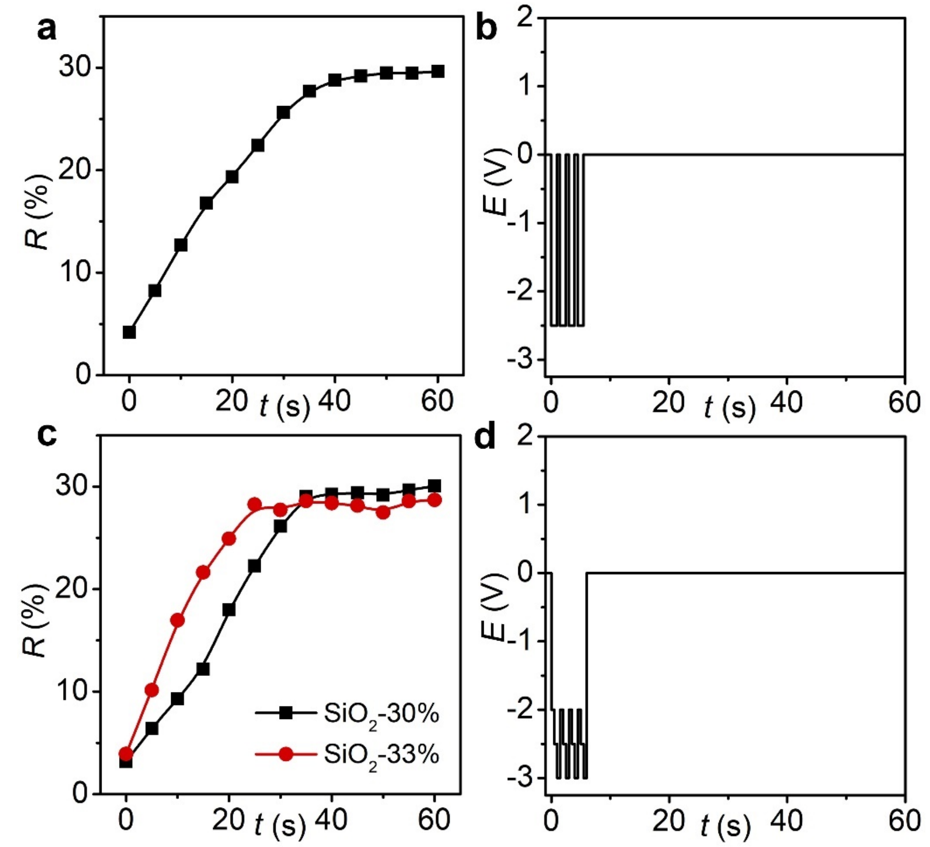

Supplementary Figure 30. Accelerating the recovery of colored state by E-field modulation. a The recovery of SiO_2/PCb-PEG-EG (30/60/5/5) ERPC under **b** an impulsive electric field, indicated by time evolution of reflection intensities. The recovery time is 32 s. **c** The recovery of two SiO_2/PCb-PEG-EG ERPCs (30/60/5/5; 33/57/5/5) under **d** a step-form electric field. The recovery times are 30 s and 22 s, respectively.

Supplementary Tables

Supplementary Table 1. The composition of all ERPCs tested in the current work.

ERPC	f_{SiO2} (%)	f_{PCb} (%)	f_{PEG} (%)	f_{EG} (%)	Test
SiO_2/PCb-PEG20000-EG	30	60	5	5	Fig. 1
SiO_2/PCb-PEG1500	30	67.5-60	2.5-10	0	Fig. 2a
SiO_2/PCb-PEG20000	30	67.5-60	2.5-10	0	Fig. 2b
SiO_2/PCb-PEG20000-EG	30	65-55	5	0-10	Fig. 2d, 2e
SiO_2/PCb-PEG20000-EG	25-35	65-55	5	5	Fig. 2g-2i
SiO_2/PCb-PEG20000-EG	30	60	5	5	Fig. 3-5

Supplementary Table 2. Evaluation of the holding of the colorless state by a low reflection intensity retaining ratio (R_{600}/R_0) or a low reflection intensity recovery rate ($\Delta R/t$) when the ERPCs were exposed to the 4.5V for 5 s and then kept at 0V for 600 s.

ERPC	f_{SiO2} (%)	f_{PCb} (%)	f_{PEG} (%)	f_{EG} (%)	R_{600}/R_0 (%)	$\Delta R/t$	Test
SiO_2/PCb-PEG1500	30	67.5	2.5	0	92.7	0.070	Fig. 2a
SiO_2/PCb-PEG1500	30	65	5	0	81.2	0.033	Fig. 2a
SiO_2/PCb-PEG1500	30	60	10	0	33.3	0.011	Fig. 2a
SiO_2/PCb-PEG20000	30	67.5	2.5	0	26.7	0.014	Fig. 2b
SiO_2/PCb-PEG20000	30	65	5	0	14.7	0.008	Fig. 2b
SiO_2/PCb-PEG20000	30	60	10	0	5.1	0.003	Fig. 2b
SiO_2/PCb-PEG20000-EG	30	65	5	0	14.7	0.008	Fig. 2d
SiO_2/PCb-PEG20000-EG	30	60	5	5	6.8	0.004	Fig. 2d
SiO_2/PCb-PEG20000-EG	30	55	5	10	6.4	0.004	Fig. 2d

Supplementary Table 3. The kinematic viscosity of the PCb-PEG mixing solvents measured at 25 °C

Solvent	f_{PCb} (%)	f_{PEG} (%)	η (cSt)
PCb	100	0	2.72
PCb-PEG1500	97.5	2.5	3.64
PCb-PEG1500	95	5	4.76
PCb-PEG1500	90	10	7.62
PCb-PEG20000	97.5	2.5	9.62
PCb-PEG20000	95	5	24.38
PCb-PEG20000	90	10	101.75

Supplementary Table 4. Evaluation of the fast recovery of the colored state by a high reflection intensity recovery ratio (R_{60}/R_0) or a high reflection intensity recovery rate ($\Delta R/t$) when the ERPCs were exposed to the -2.5V for 4 s and then kept at 0V for 60 s.

ERPC	f_{SiO2} (%)	f_{PCb} (%)	f_{PEG} (%)	f_{EG} (%)	R_{60}/R_0 (%)	$\Delta R/t$	Test
SiO_2/PCb-PEG20000-EG	30	65	5	0	98.5	0.604	Fig. 2e
SiO_2/PCb-PEG20000-EG	30	60	5	5	99.3	1.029	Fig. 2e
SiO_2/PCb-PEG20000-EG	30	55	5	10	46.8	0.281	Fig. 2e

Supplementary Notes

Supplementary Note 1. Calculation of the particle volume fraction of ERPC in the colored and colorless state

The particle volume fraction (f_{SiO2}) is an important parameter for the liquid colloidal photonic crystal, which affects the viscosity (η) of the colloidal system and the viscous retarding force applied to the particles. Therefore, it will be very helpful for the explanation of the movement or assembly behavior of particles under different conditions. It should be emphasized that the so-called f_{SiO2} refers to the particle fraction within a specific structure, such as the colloidal crystal or the disordered colloidal stacking. It does not refer to the average particle fraction of the whole colloidal system, which is a constant determined by the recipe of the bistable ERPC.

For a liquid PC with *fcc* lattice but non-close-pack structure, f_{SiO2} can be calculated by Eq (1), where *r* is the radius of the SiO_2 particles (85 nm) determined from the TEM observations, and *D* is the center-to-center distance between neighboring particles, which can be calculated by the Bragg's equation for colloidal crystals.

$$f_{SiO2} = \frac{4\times\frac{4}{3}\pi r^3}{(\sqrt{2}D)^3} = \frac{\pi}{3\times\sqrt{2}}\left(\frac{2r}{D}\right)^3 = 0.74\times\left(\frac{2r}{D}\right)^3 \quad (1)$$

In the Bragg's equation (Eq 2), λ is the reflection wavelength of the liquid PC measured by the spectrometer, *D* is the particle interspacing, and n_{eff} is the effective refractive index of the whole system. For a colloidal solution, n_{eff} can be calculated by Eq (3), where $n_{particle}$, n_{medium}, $f_{particle}$, and f_{medium} are the refractive index and volume fraction of SiO_2 particles and the surrounding medium. Here, n_{eff} is calculated to be 1.4345 according to the refractive index of SiO_2, PCb, PEG, and EG (1.46, 1.42, 1.46 and 1.43) and their dosage fractions (30%, 60%, 5% and 5%). Since the refractive index of four components are very close to each other, n_{eff} is considered as a constant in different colloidal structures to simplify the calculations.

$$\lambda = \left(\frac{8}{3}\right)^{1/2} \times D \times n_{eff} \quad (2)$$

$$n_{eff} = n_{particle} \times f_{particle} + n_{medium} \times f_{medium} \quad (3)$$

When the SiO_2/PCb-PEG-EG ERPC is in the "colored state", the SiO_2 particles are orderly arranged

in the solution with λ measured to be 541 nm. D is then calculated to be 230.9 nm through Eq (2). Eventually, the f_{SiO2} is calculated to be 29.6% by Eq (1).

When the SiO_2/PCb-PEG-EG ERPC is in the "colorless state", the f_{SiO2} should be smaller than the maximum packing fraction (64%) for a disordered particle stacking. Meanwhile, the f_{SiO2} should be larger than the particle fraction corresponding to the last observable PC structure with most blueshifted λ (453 nm). Because the ERPC loses its structural color along with the further compression and destruction of colloidal crystal structure, which causes an increase in f_{SiO2}. D for the most compressed liquid PC is then calculated to be 193.4 nm through Eq (2), and corresponding f_{SiO2} is calculated to be 50.4% by Eq (1). Therefore, the f_{SiO2} of the ERPC in the "colorless state" should be within the range from 50.4% to 64%.

Supplementary Note 2. Calculation of the relative viscosity of ERPC in the colored and colorless state

For the colloidal suspension with high particle volume fraction, the relative viscosity of the suspension (η_r) , which refers to the ratio between the viscosity of suspension and that of the solvent, can be estimated by Eq (4),[1] where f_{SiO2} is the volume fraction of the SiO_2 particles and f_{max} is the maximum packing fraction of particles. According to the reference, f_{max} is determined to be 0.64 for colloidal suspensions from monodisperse particles. Apparently, a larger f_{SiO2} leads to a larger η_r for the colloidal suspension.

$$\eta_r = (1 - \frac{f_{SiO2}}{f_{max}})^{-2.5 f_{max}} \quad (4)$$

When the SiO_2/PCb-PEG-EG ERPC is in the "colorless state", the f_{SiO2} is calculated to be larger than 50.4%, and η_r is estimated to be higher than 11.9 according to Eq (4). Therefore, the viscosity of the whole colloidal suspension is extremely high in this state, considering it equals to 11.9 folds of the viscosity of the PCb-PEG-EG mixture. Such high viscosity enhances the viscous retarding force, freezes the Brownian motion of particles, and stabilized the disordered particle arrangement.

When the SiO_2/PCb-PEG-EG ERPC is in the "colored state" or a "quasi-ordered" state, the f_{SiO2} is calculated to be 29.6%, and η_r is estimated to be 2.7 according to Eq (4). In this state, the viscosity of the whole colloidal suspension is only 2.7 folds of the viscosity of the PCb-PEG-EG mixture. As a

result, the viscous retarding force was not strong enough to freeze the particles' motions, which let them assemble into ordered structure again.

All the calculations above suggested that the switching of the stable states for ERPC are driven by the change in f_{SiO2} and η. The exertion of forward voltage quickly increases the f_{SiO2} and lock the disordered structure, while the exertion of inverse voltage decreases the f_{SiO2} and let the particles reassemble into ordered structure.

Supplementary References

1. Adamczyk, Z., Jachimska, B., Kolasinska, M. Structure of colloid silica determined by viscosity measurements. *J. Colloid Interface Sci.* **273**, 668-674 (2004).
