# Supplementary material for: Electrically responsive photonic crystals with bistable states for low-power electrophoretic color displays
Source: Nat Commun. 2022 Nov 16;13:7007. doi: 10.1038/s41467-022-34745-0 (PMC9669026; doi:10.1038/s41467-022-34745-0)
Supplement: Supplementary file 3 — Description of Additional Supplementary Information [file 41467_2022_34745_MOESM3_ESM.pdf]

Description of Additional Supplementary Information

Title: Supplementary Movie 1:

Description: Switching between 8 and 3 on numerical indicator 74

Title: Supplementary Movie 2:

Description: Switching between 8 and 6 on numerical indicator
